# An Ultrasonographic Analysis of the Deep Inferior Tendon in the Masseter Muscle: Implications for Botulinum Toxin Injections

**DOI:** 10.3390/toxins16090391

**Published:** 2024-09-11

**Authors:** Jia Shi, Chenyang Li, Jinbo Zhou, Xinyu Guo, Guo Li, Meng You

**Affiliations:** 1 State Key Laboratory of Oral Diseases & National Center for Stomatology & National Clinical Research Center for Oral Diseases, West China Hospital of Stomatology, Sichuan University, Chengdu 610041, China; jiashi0601@163.com (J.S.); xinyu_guo@stu.scu.edu.cn (X.G.); 2 State Key Laboratory of Oral Diseases & National Center for Stomatology & National Clinical Research Center for Oral Diseases & Department of Oral Medical Imaging, West China Hospital of Stomatology, Sichuan University, Chengdu 610041, China; aaakshei@163.com (C.L.); m17711384006@163.com (J.Z.); 3 State Key Laboratory of Oral Diseases & National Center for Stomatology & National Clinical Research Center for Oral Diseases & Department of Plastic Surgery and Cosmetic Dermatology, West China Hospital of Stomatology, Sichuan University, Chengdu 610041, China

**Keywords:** botulinum toxin type A injections, ultrasonographic, masseter hypertrophy

## Abstract

(1) Background: With the increasing aesthetic pursuit of facial features, the clinical use of Botulinum Toxin Type A (BoNT-A) injections for masseter hypertrophy has been on the rise. However, due to variations in masseter muscle structure and differences in clinicians’ injection techniques, blind injections may lack precision, potentially compromising treatment accuracy and increasing the risk of complications. (2) Objectives: The study aims to use ultrasonography to detail the deep inferior tendon (DIT) within the masseter muscle in a young Chinese cohort, refine its classification, analyze muscle belly thickness and variations across groups, and propose a customized ultrasound-guided BoNT-A injection protocol. (3) Methods: Ultrasound imaging was used to observe the bilateral masseter muscles at rest and during clenching. The features of the DIT were classified from these images, and the thickness of the masseter’s distinct bellies associated with the DIT types was measured in both states. (4) Results: The study cohort included 103 participants (27 male, 76 female), with 30 muscles in the normal masseter group and 176 muscles in the hypertrophy group. The DIT was categorized as Type A, B (subtypes B1, B2), and C. The distribution of these types was consistent across normal, hypertrophic, and gender groups, all following the same trend (B > A > C). In hypertrophy, Type B1 showed uniform thickness across masseter bellies, B2 presented with a thinner intermediate belly, and Type C had mainly superficial muscle enlargement. Changes in muscle thickness during clenching were noted but not statistically significant among different bellies. (5) Conclusions: The study evidences individual variation in the DIT, highlighting the importance of precise DIT classification for effective BoNT-A injections. A tailored ultrasound-guided BoNT-A injection strategy based on this classification may enhance safety and efficacy of the therapy.

## 1. Introduction

The masseter muscle is a thick, powerful muscle located in the cheek area, responsible for the chewing motion of the jaw. Originating from the zygomatic arch and inserting into the ramus of the mandible, the masseter muscle’s unique anatomical positioning and fiber orientation contribute to its crucial role in defining the facial contour and angularity of the lower face [1]. In the demographic of non-obese individuals between the ages of 20 and 40 years old, the predominant cause of noticeable mandibular angle and square-shaped face is often attributed to simple masseter hypertrophy [1]. This condition is frequently a consequence of an interplay of factors encompassing dietary habits, masticatory practices, and hereditary influences [1]. Previous studies indicate that the average width of the lower facial region in Asians exceeds that of their Caucasian counterparts [2]. Influenced by cultural prevailing aesthetics, Asians, especially females, are pursuing a more delicate and slender mandibular contour [3].

In 1994, Moore first introduced the utilization of Botulinum Toxin Type A (BoNT-A) for facial sculpting [4]. Following this, Rijsdijk et al. initiated the application of BoNT-A for addressing masseter hypertrophy in patients seeking cosmetic enhancements [5]. BoNT-A works by inhibiting acetylcholine release at the neuromuscular junction, resulting in muscular relaxation and atrophy, which consequently leads to a reduction in muscle volume and an enhancement of facial contours [6,7,8]. Due to its minimal-invasive nature, effective results, low risk, and quick recovery, BoNT-A injection has become the preferred treatment for masseter hypertrophy [9,10].

Currently, although the administration of BoNT-A is deemed to have a relatively high safety profile, the potential for complications persists due to the complex anatomy and its inter-individual variability at the injection region [11,12,13]. One common complication is paradoxical masseteric bulging (PMB) following BoNT-A injection into the masseter muscle, with reported incidence rates ranging from 0.15% to 27.3% [13,14,15]. PMB typically manifests after one week post-injection and is closely related to the anatomic variation of the masseter muscle, particularly the variation in the deep inferior tendon (DIT) located deep within the superficial part of the muscle [13,16,17,18]. The ideal injection of BoNT-A ensures its even distribution throughout the muscle to achieve adequate paralysis. However, the DIT, which divides the masseter muscle into a deep and superficial belly, may prevent the BoNT-A injected into the deep belly from diffusing into the superficial belly [16]. As a result, the superficial belly remains robust and even overcompensates during contraction, leading to paradoxical bulging.

Therefore, understanding the position and pattern of the DIT is essential for precise and adequate injection. In 2017, Lee et al. first identified the presence of the DIT within the masseter through cadaveric dissection [16]. Following this, they conducted a series of ultrasound-based studies to investigate the patterns and locations of the DIT, which formed their DIT-based injection technique [17,19]. Other research groups have used longitudinal ultrasonographic images to measure the thickness and variation of the deep and superficial bellies of the masseter muscle segregated by the DIT [20,21]. However, these studies did not adapt the measurement strategies for different types of DITs. Moreover, while the statement has been made that ultrasonographic examinations should be conducted before or during injection to assess the structural pattern of the DIT to create personalized injection plans, specific injection methods for distinct DIT types have not been proposed.

In this study, we utilized ultrasound imaging to thoroughly characterize the DIT in young Chinese individuals by including a larger sample size. The focus of the research was on refining the classification of DIT, dividing the deep and superficial bellies based on the characteristics of different types of DITs in transverse images, and measuring their thickness, respectively. Moreover, by comparing the DIT classification and dynamic change characteristics between different groups, we proposed a safer and more efficient ultrasound-guided DIT-based personalized BoNT-A injection protocol.

## 2. Results

### 2.1. Clinical Profiles

The study cohort included 103 subjects with 27 males (age range 21–27 years old; mean age 22.52 ± 1.74 years old) and 76 females (age range 19–35 years old; mean age 23.93 ± 2.67 years old). In total, 206 masseter muscles were examined. Based on the established grouping criteria [14,22,23], there were 30 cases classified into the normal masseter muscle group (with an average thickness of 12.98 ± 0.58 mm for males and 11.40 ± 0.56 mm for females during clenching) and 176 cases classified into the masseter hypertrophy group (with an average thickness of 16.54 ± 1.33 mm for males and 14.82 ± 1.88 mm for females during clenching).

### 2.2. Classification of DIT Patterns

Ultrasound images of the DIT were obtained for all subjects on both the left and right sides, with the DIT observed in all ultrasound images as a bright echogenic band. In the transverse ultrasound images, the DIT courses through the superficial masseter.

**Figure 1:**
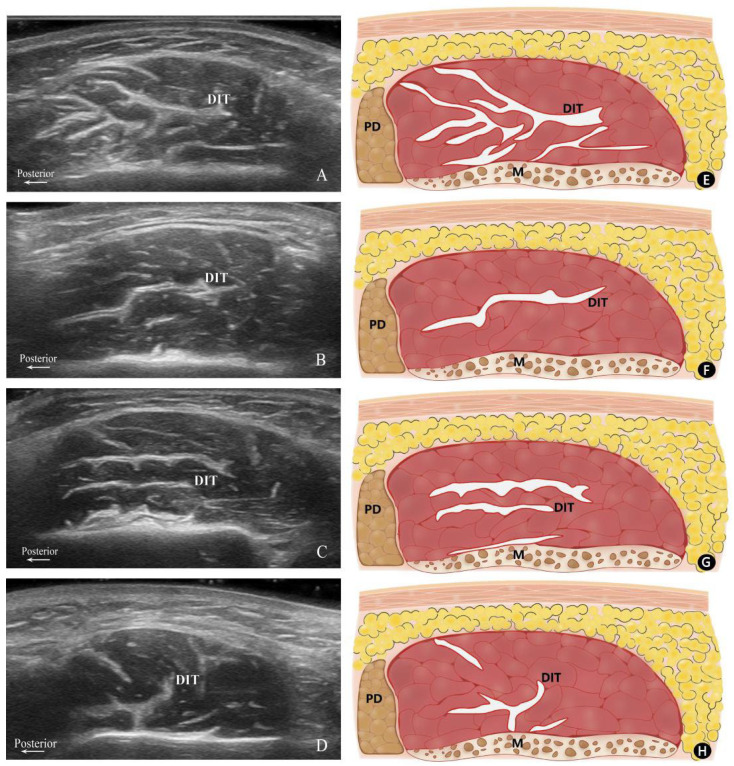
Transverse ultrasonography images and their corresponding schematics. Ultrasonography images of Type A (**A**), B1 (**B**), B2 (**C**), and C (**D**) along with their corresponding schematics: (**E**) Type A, (**F**) Type B1, (**G**) Type B2, and (**H**) Type C. P, posterior; M, mandible; PD, parotid gland; DIT, deep inferior tendon.

Based on their configuration and distribution within the masseter muscle, the patterns of the DIT were classified into three distinct types. Type A refers to the muscle being longitudinally divided into anterior and posterior portions, with the posterior portion exhibiting a complex and irregular course of the DIT. Type B denotes the DIT partitions the muscle in a transverse manner, where Type B1 is characterized by the muscle being separated into superficial and deep bellies by a single transverse DIT, and Type B2 is distinguished by two transverse DITs segmenting the muscle into superficial, middle, and deep bellies. Type C signifies the presence of a distinct DIT coursing both longitudinally and transversely, with the transverse DIT dividing the muscle into superficial and deep bellies. However, the longitudinal DIT is too short to separate the masseter into anterior and posterior partitions (Figure 1).

The study revealed that among the male subjects, the most prevalent DIT pattern was Type B (29 out of 54, 53.7%), with a higher occurrence of subtype B1 (24 out of 54, 44.4%), followed by Type A (19 out of 54, 35.2%), and Type C being the least common (6 out of 54, 11.1%). Among female participants, the proportion of DIT patterns was distributed as follows: Type A at 39.5% (60 out of 152), Type B at 54.6% (83 out of 152), and Type C at 5.9% (9 out of 152). The most common DIT structural pattern in both males and females was subtype B1, with no statistical difference in the distribution of types between male and female subjects (Table 1).

When comparing the DIT structural patterns between the normal masseter group and the masseter hypertrophy group, subtype B1 also emerged as the most common pattern in both groups, followed by Type A. Type C was also the least observed pattern, regardless of whether the subjects were in the normal or hypertrophic group. There was no statistical difference in the distribution of types between the normal masseter group and the masseter hypertrophy group (Table 2).

### 2.3. DIT-Based Analysis of the Masseter Muscle Thickness

**Figure 2:**
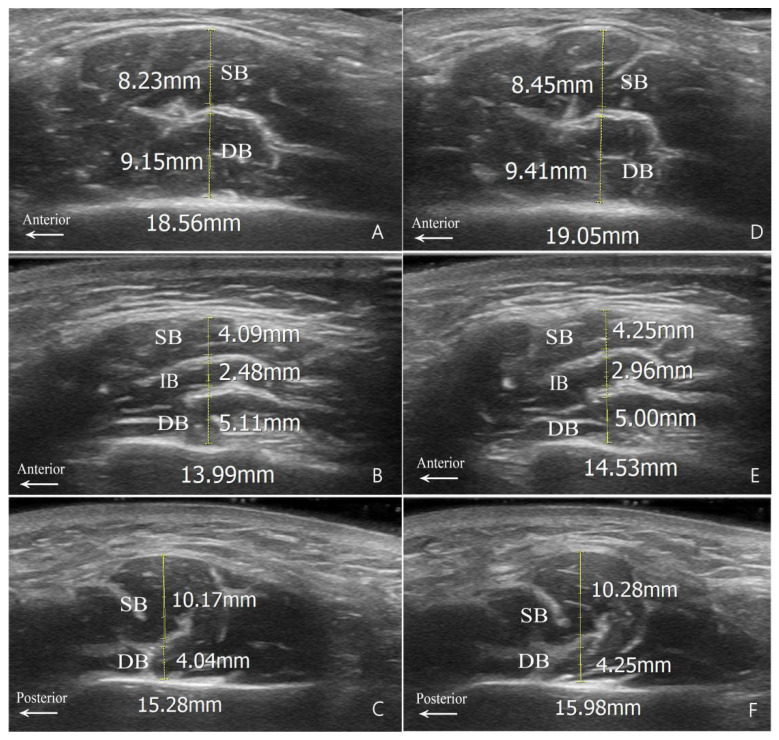
Measurement diagrams for muscle belly thickness. (**A**,**D**) Type B1; (**B**,**E**) Type B2; and (**C**,**F**) Type C. Relaxed state images are shown on the left (**A**–**C**); clenched state images are shown on the right (**D**–**F**). SB, superficial belly; IB, intermediate belly; DB, deep belly; DIT, deep inferior tendon.

Since the superficial portion of the masseter muscle in Type A is longitudinally rather than transversely separated by the DIT, the measurements based on the DIT in this study were confined to the other types (Figure 2). The measured thicknesses of these muscle bellies in the hypertrophy group, in both relaxed and clenched states for Types B and C, are presented in Table 3.

The ICC between the two independent examiners demonstrated a good agreement for Type B measurements (all ICCs > 0.7). However, for Type C, the ICC values for the thickness measurements of the superficial and deep bellies ranged between 0.43 and 0.65, denoting moderate consistency (Table 4).

Within the B1 structural pattern, no statistically significant difference was observed between the average thicknesses of the superficial and deep bellies, either in relaxed or clenched states (Relaxed *p* = 0.264; Clenched *p* = 0.352). For Type B2, significant differences were found between the thickness of the intermediate and both the superficial (*p* = 0.001) and deep (*p* = 0.008) bellies in the relaxed state, though no distinction was noted between superficial and deep measurements (*p* = 1.000). The results in the clenched state were similar to those in the relaxed state. In the Type C structural pattern, significant variations in thickness between the superficial and deep bellies were consistent in both relaxed and clenched states, with the superficial belly being thicker (Relaxed *p* = 0.019; Clenched *p* = 0.016). Therefore, the statistical analysis reveals that in Type B1 masseter hypertrophy, there is no discernible disparity in the thickness between superficial and deep bellies. In contrast, Type B2 masseter hypertrophy is characterized by thickness predominantly in both the deep and superficial bellies, with the middle belly being the thinnest. For Type C masseter hypertrophy, the enlargement is primarily in the superficial belly. Moreover, upon comparing the belly measurements with the normal group, an increased thickness in both deep and superficial bellies of Type B1 were found in the hypertrophy group, under both relaxed and clenched states.

When the masseter muscle shifted from a relaxed to a clenched state, alterations in thickness were noted across all bellies (Table 3 C-R). However, there was no statistically significant difference in this change observed between the superficial and deep bellies in Types B1 and C (P1 = 0.711, P2 = 0.091), nor among the superficial, intermediate, and deep muscle layers in Type B2 (*p* = 0.662).

## 3. Discussion

The DIT within the masseter muscle plays a vital role in the formulation of BoNT-A injection treatment strategies for masseter hypertrophy. Prior research has primarily focused on the fundamental anatomical aspects of the DIT, such as its precise location and distribution within the masseter. In contrast, the current study included a larger sample size and adopted more precise measurements to characterize the DIT, proposing a new classification to guide clinical injections. In addition, we emphasize the use of ultrasound technology for accurately identifying the DIT types, which enables medical practitioners to pinpoint the optimal injection sites and depths, thereby improving treatment efficacy and safety. Furthermore, our findings provide a theoretical foundation for personalized treatments based on the DIT types. This highlights the significance of comprehensive consideration of the DIT through ultrasound examination in achieving optimal treatment outcomes for masseter hypertrophy.

Anatomical studies reveal that the masseter muscle consists of three layers: the superficial, intermediate, and deep layer, each exhibiting distinct contraction patterns [24]. The deeper the muscle layer, the higher its inferior insertion point. The superficial layer—originating from the zygomatic process of the maxilla and the anterior two-thirds of the zygomatic arch’s inferior border, and inserting into the inferior part of the mandibular angle and the outer surface of the ramus—is typically the main contributor to masseteric hypertrophy. Thus, the superficial masseter is the principal target for BoNT-A injections.

It is essential to note that the risorius muscle, which originates from the fascia, is positioned anterior to and overlying the superficial masseter. Consequently, the injection site should be positioned neither too anteriorly nor too superficially to prevent impairment of the risorius muscle’s function, which could adversely affect the natural contraction of the corners of the mouth and lead to asymmetric facial expressions [25]. Thus, in line with current clinical consensus, injections should be administered at an appropriate depth [10,26,27,28]. To maximize BoNT-A’s efficacy, injections should target the neuromuscular junction area, where the drug could be taken up by the presynaptic membrane of motor neurons [29,30,31]. The branches of the masseteric nerve are primarily located in the middle to lower part of the masseter, and the most prominent region of the masseteric prominence corresponds to the central distribution area of these masseteric nerve branches [32,33]. Consequently, the most prominent point of the masseter is an ideal site for BoNT-A injections [34,35].

The DIT has been identified as a consistent structure in the masseter, positioned deep in the lower third of its superficial layer, corresponding to the ideal injection site as previously discussed [32,33,34,35]. Previous studies that employed ultrasound to observe the DIT conducted dynamic scans within the region from the mandibular angle to the tragus-commissure line, which is also the injection area [17]. In the present study, ultrasound images were obtained at the site where the DIT structure is most clearly visible, which is also within the injection area. Thus, the resulting DIT classification and measurements derived from these images hold clinical significance for guiding injections. The objective of the BoNT-A injection method for the treatment of masseter hypertrophy is to ensure an even distribution of the toxin within the masseter, followed by the achievement of consistent muscle atrophy. Nevertheless, recent research outcomes clearly show that the DIT can further subdivide the superficial layer of the masseter into various muscle bellies, acting as a barrier that confines the injected toxin to a specific belly, thus preventing its uniform distribution across all bellies [14,16,19,36,37]. This indicates that the structure and distribution of the DIT barrier have a direct impact on the effectiveness of the injection therapy and are closely associated with adverse reactions such as PMC [16,38].

Due to the significant impact of the DIT on BoNT-A injections, previous studies have investigated its morphology and distribution for better treatment efficacy. In 2017, Lee et al. dissected the bilateral masseter muscles of 44 cadavers, dividing the muscle into six regions by equally partitioning it both vertically and horizontally. The DIT was subsequently classified into three types based on its coverage of the lower masseter: Type A, covering the anterior two-thirds; Type B, covering the posterior two-thirds; and Type C, encompassing the entire lower region [16]. Type C was most common in Korean cadavers, while Type A was prevalent in Thai cadavers. Building on this work, in 2019, Lee et al. conducted an ultrasound-based study on 30 subjects, refining the classification of the DIT into compartmentalized, transverse, and longitudinal types, based on transverse ultrasound images with the masseter in a relaxed state [17]. The study revealed a gender-specific distribution, with the compartmentalized type being predominant in the right masseter of males, and the longitudinal type in females. However, a subsequent study by Li et al., utilizing the same classification methodology, reported the compartmentalized type as the most commonly observed across all subjects [21].

In this study, we further refined the DIT classification in ultrasound images according to a more detailed description and analysis of its pattern and course by increasing the sample size and analyzing the images acquired at both relaxed and clenching states. We found that there was no statistically significant difference in the composition of DIT types between two genders. This finding aligns with previous research, suggesting that the internal structural characteristics of the masseter muscle appear to be independent of gender [17,39]. Moreover, we found the distribution of DIT types was also similar between the normal and hypertrophy masseter groups.

By analyzing transverse ultrasound images, we classified the DIT into three main types. Type A is similar to the compartmentalized type in Lee’s study, where the DIT completely compartmentalizes the superficial posterior portion of the masseter, exhibiting a complex course. Type B, akin to Lee’s transverse type, primarily features a transverse course, but we further divided it into Type B1 (single transverse tendon) and Type B2 (two transverse tendons). Type C consists of both longitudinal and transverse DITs, with Lee’s longitudinal type falling under Type C. However, our collected images revealed that the longitudinal DITs often accompanied transverse branches, with both longitudinal and transverse DITs originating from a single point or different points. Within our samples, Type B was the most common morphology, particularly Type B1, followed by Type A, while Type C was relatively less frequent.

Muscle thickness is often correlated with muscle strength. Thus, the clinicians normally make the dose adjustments based on the hypertrophy of the masseter muscle. Excessive doses of BoNT may result in diffusion to adjacent muscles, causing unwanted paralysis [16,38] and a range of complications (such as dysphagia and temporomandibular joint pain) [15,40]. Moreover, it has been revealed that the human system produces antibodies when exposed to excessive BoNT-A, which may impair its effectiveness in future treatment [41,42]. To achieve personalized dose injections based on the DIT, Wang et al. assessed the morphological changes in the masseter muscle of 15 subjects under relaxed and clenched conditions using ultrasound [20]. The study found that the deep belly was thicker in both relaxed and clenched states based on the anatomical segregation of the DIT in longitudinal ultrasound images. However, when Li et al. used the same measurement method on 42 participants, they reached an opposite conclusion [21].

Contrary to previous studies that measured bellies based on the DIT in longitudinal ultrasound images, our study was conducted on transverse ultrasound images. Transverse ultrasound images enable classification based on the course of the DIT and measurement of muscle bellies divided by DIT type, allowing injection methods to be designed specifically for DIT types. Our measurements revealed that the precise thickness of muscle bellies varied among patients with different DIT types. Type B1 demonstrated similar thickness in both the superficial and deep muscle bellies. Conversely, Type B2 had the thinnest intermediate muscle belly, whereas Type C displayed a thicker superficial muscle belly. Thickness changes in muscle bellies separated by the DIT from relaxed to clenched states were not statistically significant. The ICC results indicated that the measurements for Type B DITs had good consistency, while the ICC for Type C showed slightly lower consistency. The discrepancy can potentially be attributed to a minor deviation in the transverse DIT shape in Type C (in contrast to the relatively straight DIT in Type B), as well as the existence of longitudinal branches. Therefore, it may have led to a discrepancy in the determination of the measurement point between the researchers.

Utilizing masseter muscle belly thickness ratios, we can allocate BoNT-A doses more effectively, achieving better treatment outcomes with smaller doses. For Type A, characterized by complete posterior masseter separation and intricate branching, it is recommended to pay more attention to this area during the injection process. Therefore, it is advisable to conduct the injections with precision under the guidance of ultrasonography to facilitate an equal distribution of the toxin, optimizing its spread in this area with complicated DIT branches.

To ensure accurate injection, an ultrasound examination needs to be conducted. The distinguished layering of the masseter muscle in Type B allows for precise injections and dose allocations based on differences in muscle belly thickness. This study’s findings indicated that the optimal dose distribution ratio for Type B2’s superficial, intermediate, and deep belly is 2:1:2. Type C is relatively uncommon, and the layering of the masseter belly is less apparent compared to Type B. To minimize the risk of inaccuracy for Type C injections, it is advisable to conduct multiple observations and measurements. In cases of necessity, injections can be administered under ultrasound guidance to ensure that the BoNT reaches all muscle bellies.

**Figure 3:**
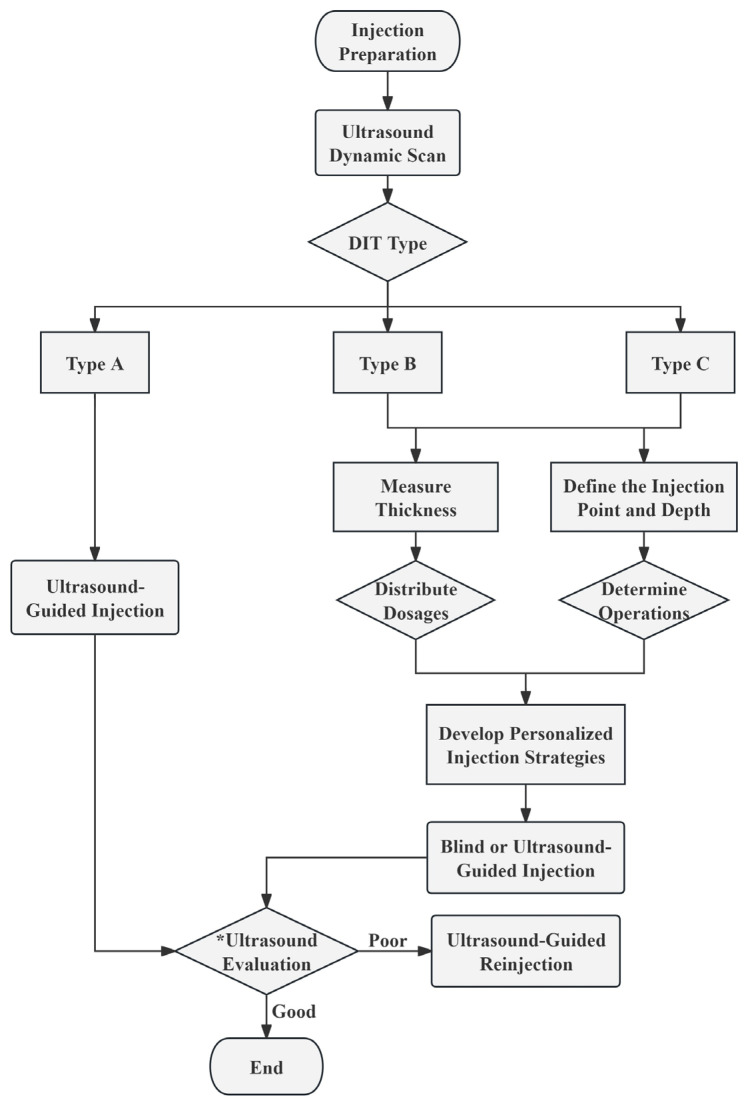
The recommended procedural flowchart for injecting BoNT-A. * Ultrasound Evaluation: Evaluate the injection effect by ultrasound at 1, 2, 3, and 4 weeks post-injection. Throughout the 4 subsequent assessments, observe the condition of the muscle fibers and dynamically monitor whether the masseter muscle contracts evenly. At the 4-week mark, compare the thickness of the masseter muscle before and after the injection. Good Injection Effect: By the 4th week, the masseter muscle thickness is reduced to achieve the desired injection outcome, with no complications. Poor Injection Effect: Ineffective injections or complications may arise within 4 weeks. DIT, deep inferior tendon.

Based on the preceding research findings, we propose the following injection strategies tailored to DIT classifications and measurement data, intended for reference in subsequent clinical studies and practice. The detailed procedural flow is depicted in the figure below (Figure 3).

Although our research has provided new insights into the treatment of masseter muscle hypertrophy with BoNT-A injections, there are still some limitations and unexplored areas. Firstly, our study is a cross-sectional study aimed at proposing an ultrasound-guided, precise, personalized BoNT-A injection method based on the DIT for better treatment of masseter muscle hypertrophy. Ultrasound-guided BoNT-A injection techniques are becoming increasingly common in clinical practice. This approach enhances injection precision while effectively reducing patient discomfort and the risk of complications [43,44]. However, further clinical trials are necessary to evaluate whether the outcomes of injections performed using this strategy can enhance treatment safety and efficacy, as well as increase patient satisfaction. Long-term efficacy assessments should also be conducted, such as evaluating the treatment durability and possible complications in different types of DIT patients. Additionally, exploring the potential applications of DIT classification in the treatment of other oral and maxillofacial conditions may provide solutions to a wider range of clinical problems. Furthermore, considering the multifactorial etiology of masseter muscle hypertrophy, future research could explore comprehensive treatment methods guided by DIT classification.

## 4. Conclusions

Based on the findings of our study, we propose a personalized treatment protocol for masseter hypertrophy that is founded on the ultrasound evaluation of the DIT. Through a detailed examination of ultrasound images, we are capable of precisely classifying the DIT, which enables us to create a customized BoNT-A injection strategy. It is our recommendation that clinicians should initiate the treatment of masseter hypertrophy by utilizing ultrasound to conduct a detailed classification and analysis of the DIT, and then construct personalized treatment plans based on these detailed findings. This personalized injection strategy, leveraging the accuracy of ultrasound imaging and the intricate architecture of the DIT, holds the promise of delivering a more refined, targeted, and efficacious therapeutic outcome for patients.

## 5. Materials and Methods

### 5.1. Subjects

Volunteers were publicly recruited in a hospital from November 2023 to February 2024; clinical and ultrasound examination results of bilateral masseter muscles were recorded for each individual.

The study’s inclusion criteria consisted of individuals aged 18 to 40 of any gender, while the exclusion criteria comprised individuals undergoing orthodontic treatment, those diagnosed with oral and maxillofacial tumors, individuals who had previous plastic surgery, or those who had received a BoNT injection within the preceding 6 months.

Before participating in the study, a signed informed consent form was obtained from each volunteer. All examination procedures in this study were conducted in accordance with the Declaration of Helsinki of the World Medical Association. The study was approved by the Institutional Review Board (WCHSIRB-CT-2024-286).

### 5.2. Ultrasound Examination and Analysis

#### 5.2.1. Ultrasound Examination

Participants were positioned supine on the practitioner’s right side. The initial step involved palpation of the masseter muscle, both at rest and with teeth clenched, to identify its approximate extent. A real-time 2D B-mode ultrasound device (Mindray Resona 5) equipped with a high-frequency (12.0 MHz) linear transducer was utilized to determine the precise boundaries of the masseter muscle and to acquire images.

The scanning region encompassed the anterior and posterior borders of the masseter muscle and the segment between the lower edge of the mandible and the line connecting the tragus to the corner of the mouth, where the thickest plane of the masseter muscle and the common clinical injection sites are often located. The ultrasound probe was carefully placed on the aforementioned scanning region perpendicular to the skin surface, ensuring no compressive deformation occurred in the area being measured. The horizontal scan was conducted by placing the probe parallel to the lower edge of the mandible, moving within the area below the imaginary line drawn from the corner of the mouth to the tragus.

Prior to the formal study, a preliminary experiment was conducted wherein ultrasound images of the masseter muscles were obtained from 15 subjects, resulting in a total of 30 images. Through a series of iterative tests, a robust methodology for ultrasound image acquisition was developed, ensuring both the accuracy of image collection and the overall quality of the images. To maintain consistency and reduce potential errors, all imaging procedures were conducted by the same practitioner (ZJB), who has over 10 years of clinical experience in US examination. Bilateral masseter muscle images were collected for each subject.

#### 5.2.2. US-Based Analysis of the DIT

Starting from the most relaxed state of the masseter muscle, the process of clenching tightly and then relaxing again was repeated twice. The DIT was imaged at both muscle relaxation and contraction states. The dynamic change during the process was also recorded.

In the acquired transverse sonograms, (1) the morphology and structure of the DIT were observed for classification and (2) the thicknesses of both the superficial and deep belly of the masseter muscle were measured in the muscle relaxation and contraction states. The measurement tasks were carried out by two examiners independently, followed by an inter-examiner consistency analysis.

#### 5.2.3. US-Based Analysis of the Masseter Muscle Thickness

Participants were instructed to clench their teeth and contract the masseter muscle in order to facilitate the practitioner in locating the thickest part of the masseter muscle, which was then measured. Subsequently, they were asked to relax the masseter muscle, and the muscle thickness was measured at the located plane in the relaxed state.

### 5.3. Statistical Analysis

The normality of the data was assessed employing the Kolmogorov–Smirnov test. The inter-examiner consistency was tested using the intraclass correlation coefficient (ICC). The variations in muscle belly thickness were expressed as the proportionate amount of change in the thickness of each muscle belly between the clenched and relaxed states. The Friedman test and paired *t*-tests were then used in the analysis. The statistical analysis was carried out using IBM SPSS Statistics software, version 27.0, with differences deemed statistically significant at a *p*-value less than 0.05.

## Figures and Tables

**Table 1:** Distribution of DIT Types Between Genders.

	A	B1	B2	C	Total
Male	19	24	5	6	54
Female	60	70	13	9	152
Total	79	94	18	15	206

**Table 2:** Distribution of the DIT Types Between Groups.

	A	B1	B2	C	Total
Normal group	12	17	1	0	30
Hypertrophy Group	67	77	17	15	176
Total	79	94	18	15	206

**Table 3:** Measurement Results of Type B and Type C Based on DIT in Hypertrophy Group.

DIT Classification	State	Masseter Muscle	Superficial Belly	Intermediate Belly	Deep Belly
B1	Relaxed	0.912	0.861	-	0.821
	Clenched	0.987	0.836	-	0.790
B2	Relaxed	0.966	0.871	0.802	0.911
	Clenched	0.982	0.821	0.714	0.784
C	Relaxed	0.990	0.540	-	0.650
	Clenched	0.986	0.426	-	0.482

Data are mean ± SD values.

**Table 4:** Results of ICC Analysis.

	B1 (77)			B2 (17)			C (15)		
	Relaxed	Clenched	C-R	Relaxed	Clenched	C-R	Relaxed	Clenched	C-R
Superficial belly	6.70 ± 1.49	7.24 ± 1.65	0.29 ± 2.27	4.82 ± 1.19	5.24 ± 1.32	0.42 ± 0.58	7.23 ± 1.90	7.71 ± 2.06	0.48 ± 0.54
Intermediate belly	-	-	-	2.88 ± 0.80	3.00 ± 0.76	0.13 ± 0.58	-	-	-
Deep belly	6.41 ± 1.44	6.97 ± 1.58	0.28 ± 2.61	4.32 ± 1.10	4.90 ± 1.16	0.58 ± 0.72	5.23 ± 1.57	5.54 ± 1.54	0.31 ± 0.52

## Data Availability

Due to privacy and ethical restrictions, the data is unavailable.
